# Society Proceedings

**Published:** 1903-03

**Authors:** 


### SOCIETY PROCEEDINGS.

### IOWA STATE VETERINARY MEDICAL ASSOCIATION.

OFFICIAL REPORT OF THE PROCEEDINGS OF THE FIFTEENTH ANNUAL MEETING HELD AT CEDAR RAPIDS, IOWA, JANUARY 14, 15, 1903.

### FIRST DAY—*Morning Session.*

THE meeting was called to order at 10 A.M. in the rooms of the Commercial Club by the President, Dr. J. I. Gibson, of Denison.

The Secretary announced the system of card registration instead of roll-call.

The address of welcome was made by Mayor C. D. Huston, of Cedar Rapids, as follows:

MR. CHAIRMAN AND GENTLEMEN: I deem it an honor as the Chief Executive of the Parlor City to extend the official greetings to the members of the Iowa State Veterinary Medical Association. You are expected to have a good time—the only kind of time observed on convention days in this city when friends are to be entertained. You can go all the gaits if you choose and make a record. In other words, it is a free-for-all and none barred. And, after you have finished your labors and returned to your respective homes, it is our hope that it will be with pleasant recollections of our city and citizens.

We have the biggest city marshal in the entire country, and the most tender-hearted, too. It has been said of him, and it might truthfully be said of some other of us city officials, that in his boyhood days he was so tender-hearted that he never overloaded a "saw-horse;" so he makes a good, humane officer as well as police official. You will be under his special care and protection during your stay, and, should any of your number become lost or strayed, just telephone the city marshal and you will be found—not fined.

Every head of a family in our city is the possessor of a horse of some description. It may be a pacer or a trotter, and, if not one of those, it is sure to be a "hobby" or a "clothes-horse." But they are all interested in the veterinarians. Those who have but a clothes-horse are just as much interested in you gentlemen as the owners of the fine steppers, because they rely upon some one of your number to inspect the milk and butter fats that go into the home for use of the infants as well as the adults, and they look to you to have framed and enforced such laws as are necessary to prohibit the selling or offering for sale of any impure or adulterated foods.

I heard a horse story once—the title rôle being played by a mule. An Englishman came to this country for a hunt, choosing Missouri as the base of operations, presumably for the reason that when he returned to London he could say to his friends, "I am from Missouri; you will have to show me." He had as his guide a citizen who had been born near Penobscot, Me., and who had served time in selling wooden hams and watering dried apples. The Yankee had trained a mule so that when he tickled it in the flank it would sit down. They started upon the hunt, and after traversing the road a short distance, the guide touched the mule in the flank and it sat down. "What's the matter with the mule?" inquired the Englishman. "Nothing the matter with the mule," was the reply, "he's a setter; don't you see he is setting that rabbit," as he pointed out bunny who was scampering away. Passing along a little farther the whip was applied to the flank of the mule and it again sat down. The Englishman espied a pheasant, bagged the game, and then opened negotiations for the purchase of so intelligent and valuable an animal. After much dickering the Englishman's fine saddle-horse and trappings and one hundred dollars in coin of the realm were exchanged for the mule. Hunter and guide exchanged mounts and passed on. In a short time they came to a stream of water, and the mule, lagging somewhat, was urged on by the use of the spur, and he sat down. "What's the matter with the mule, now?" roared the Englishman. "Nothing, nothing at all," responded the Yankee, "he sets just as well for suckers as he does for game or birds."

Now, my friends, you may not know it, but it is a fact that you are just six miles from the healthiest city in the United States, according to the last census report, partly due, no doubt, to the vigilance of meat and milk inspectors, who are experienced and respected veterinarians. Marion is the capital of Linn County, and besides being noted as a health resort, is also noted as being the home of several horse-fanciers who are national characters. They will be over here to-morrow, and, with some of our own horse-fanciers, will exhibit a goodly number of beautiful coachers, park horses, single and double drivers, equal if not superior to any you have ever seen.

I am requested by the directors of the Poultry Show, now on at the Auditorium, to extend you an invitation to inspect the magnificent exhibit to be seen there, and no one can afford to miss it.

The great cereal plant, which furnishes breakfast for the world, and the biggest institution of the kind in the world, is worth a visit, as is also the Sinclair packing plant. Besides numerous other industries we have three of the largest pump factories in the West, which furnish our city wind and the population of the entire northwest with pumps and pipe—and this is no pipe-dream, either.

Then our business men want to give you the glad hand and make your stay so pleasant that you will certainly desire to, if you do not actually vote to make Cedar Rapids your permanent headquarters for all your State meetings.

Response to the address of welcome was made by Dr. J. I. Gibson.

The President delivered his address, as follows:

MR. VICE-PRESIDENT, OFFICERS AND MEMBERS OF THE IOWA STATE VETERINARY MEDICAL ASSOCIATION: I greet you at this fifteenth annual meeting, and wish you all the compliments of the season.

As each new year in the history of our Association is ushered in it brings to us and our profession new obligations and greater responsibilities. We live in an age of wonderful progress. The world is rapidly growing brighter and better. New methods are constantly coming in vogue in all commercial and professional pursuits. We discard many of the best methods of our fathers as worthless or impracticable. In this double-quick onward march of twentieth century civilization I trust our profession may always be found occupying a position in the foremost ranks.

*The Past and Present.*—It is amusing as well as instructive to compare the conditions of fifty years ago with those of the present time. How ridiculous some of the methods of those times appear when viewed in the light of the present. Few if any of the live-stock owners of to-day would permit a veterinarian to apply the plank-and-sledge-hammer treatment to a case of "lock-jaw," or the gimlet and turpentine to a case of "hollow-horn," or the knife and pepper and salt to a case of "wolf in the tail," or to introduce into the mouth of a ruminant a pork-rind or other artificial cud to relieve the patient and reinstate the suspended process of rumination. And yet the older citizens recall the time when the above-mentioned methods were very generally practised. Nowadays the owner of live-stock requires of the veterinarian better medical and surgical treatment of his animals than the medical profession was qualified to practise upon his grandfather. The improved and finely-bred animal of to-day requires much more humane and scientific treatment than did his coarser-bred ancestors. The breeder of to-day possesses a much larger fund of knowledge concerning the principles of breeding and feeding than did his fathers.

Veterinary medicine and surgery have made wonderful progress in the past fifty years. The medical profession has made, I might say, greater advancement in the past fifty years than our profession. It seems almost incredible, but it is nevertheless a fact, that asepsis and the use of antiseptics were neither known nor understood by the medical profession of fifty years ago. The army surgeons during the War of the Rebellion used a solution of potassium permanganate for the cleansing and healing of wounds, and found it an apparent aid to the healing process, but they did not know how or why it was. Some of them were particular to have and use clean instruments from a natural tendency to cleanliness, while none of them knew or understood the necessity for or the theory of sterilization of hands, instruments and all appliances and surroundings in order that good results might attend all their efforts in surgery. They looked upon suppuration and sloughing as indications of healthy progress in all wounds, but to-day if suppuration follows surgical interference, it is at once positive evidence of septic infection or lack of sterilization and is invariably charged to the negligence or carelessness of the operating surgeon or his assistants.

Aseptic results may be, and frequently are, attained at the present time in veterinary surgery, notwithstanding the fact that the surroundings favor the infection of all wounds in our patients, but fifty years ago such results were accidental in connection with human surgery and entirely beyond the conception of the human surgeon.

The older surgeons of to-day tell us they distinctly remember how their professors warned them against entering the peritoneal cavity, with the assurance that it meant almost certain death to the patient, but these same surgeons have found it necessary scores and hundreds of times to ignore the teachings of their college days, and have in so doing saved many lives that would certainly have been lost had they treated the patients as taught by the learned professors of but a generation past.

In the field of sanitation and prevention of disease the advancement has been even greater than in the art of healing. When I was a boy the people feared smallpox, diphtheria, and scarlet fever more than they now dread the so-called plagues—cholera, bubonic plague, and yellow fever; and well they might, for in those days, in the mildest outbreak of smallpox, the death-rate was about 25 per cent., as compared with 1 per cent. in recent outbreaks. In those days diphtheria very often proved fatal to whole families, while to-day 95 per cent. of diphtheria patients recover. In the treatment of these two diseases we have modern specifics, viz., vaccine and antitoxin. The thorough and systematic vaccination of a people, young and old, will, in a few generations, make that people immune to the virus of smallpox, and yet there are people abroad in our land who condemn and attempt to ignore vaccination. They are like the infidel born in a Christian land and reared by a godly mother.

Vaccination belongs to the practice of our profession to as great an extent nowadays as it does to the medical profession. Through vaccination and inoculation with immunizing serums we protect the owners of live-stock from greater losses than through all other lines of treatment and practice. We do not hesitate to assure the owner against losses from anthrax, black-leg, tetanus, Texas fever, malignant œdema, and, through the good work now being done by Drs. Pearson and Gilliland, of Pennsylvania, we hope soon to be able to assure him against loss from the spread of tuberculosis. This antituberculosis vaccination when perfected will prove the greatest blessing ever conferred upon the live-stock owners of the country, as well as a safeguard to human life. We wish these gentlemen final success in this great work for the protection of animal life and the preservation of the public health.

We noticed recently the announcement by a French scientist of the discovery of an anti-whooping cough serum, which he asserts will reduce the course of the disease to a period of not longer than one week, and prevent all spasms and paroxysms. This, if true, is one of the greatest blessings ever conferred upon mankind, especially in infancy. When we stand aside and view the rapid progress made year after year we are led to believe that nothing that can be imagined is too difficult to be brought to pass.

*The Future of the Profession.*—We might ask, What has the future in store for us and our profession in the State and nation? I feel assured that the signs of the times indicate marked advances for the veterinary profession in the near future. The great increase in the production of pure-bred stock of all breeds in the country (in which Iowa leads all other States) means a proportionate increase in the demand for qualified veterinarians. The breeder of pure-bred stock requires more of his veterinarian than does the owner of common scrub animals. He not only requires that he shall be well qualified to administer the best medical aid and to perform the most scientific surgery, but further, that he shall be qualified to instruct and to advise as to the best methods of breeding and feeding, and all other things pertaining to animal husbandry.

With all the recent improvements in the courses taught in veterinary colleges I sometimes fear the veterinarians of to-day are not fully equipped to meet the demands now made upon them. It seems to me our colleges should take the initiative in the matter and at once establish thorough courses in animal husbandry and live-stock judging. These branches properly belong to veterinary science, and every veterinarian should be expert in them. The natural tendency on the part of the breeder and feeder is to go to his veterinarian for all advice and counsel pertaining to the best methods of breeding and feeding; and the veterinarian should be qualified to advise him in the way that will bring the best results. He should also be master of the science of breeding, and able to select the proper sire and dam to produce the desired progeny.

There is an increasing demand for this kind of veterinarians to superintend breeding farms throughout the country. How many of the veterinarians present are owners and breeders of pure-bred stock of any breed or species? Every one of you should own and breed some choice animals, and thus by practical example prove your fitness to advise others in breeding.

The judging of live-stock, which is naturally the work of the veterinarian, has been of late years in this country conceded to the professors of agriculture and animal husbandry and their graduates. Many of these men are, indeed, experts, but none of them have the advantages that accrue to the veterinarian in this work on account of his thorough knowledge of the formation and function of all parts of the animal. The veterinarian in all countries excepting the United States is recognized as the proper person to judge in the show ring, and is generally made chief judge or referee at live-stock exhibitions. I for one am in favor of the judging clinics at our meetings and a course in judging at all our colleges, and the final reinstatement of the veterinarian as judge in the show ring.

*Veterinary Sanitation.*—I feel that I cannot emphasize too strongly the importance of veterinary sanitation: first, as it applies to the protection of the life and health of our domestic animals; and, secondly, on account of its effect upon human life and happiness. Just now we are confronted with a serious problem in the introduction within our borders of one of the cattle plagues of Europe—foot-and-mouth disease. If our fair State should become infected with this disease it would mean a loss of millions of dollars to our people. Why are we resting so peacefully under these circumstances? Because Uncle Sam's army of veterinary sanitarians, under the command of that peerless general, Dr. D. E. Salmon, Chief of the Bureau of Animal Industry, has promptly met the foe and thrown its invincible lines around the enemy, and is waging a war of sure and speedy extermination. For this reason we are not fearing the invasion of our State by the dread disease. As a result of organized veterinary sanitation in States and nation all contagious and infectious diseases of animals are on the decrease.

The greatest problem in veterinary sanitation yet before the civilized world is the eradication of tuberculosis from our herds. It is well for our country that the determination to control tuberculosis has become so marked before the disease gets the foothold here it has in European countries. The discovery of the antituberculosis vaccine should prove the greatest aid to the control of the disease yet known. Its use will protect all additions to an infected herd by birth or purchase and make the final purification of the herd much easier than any other method suggested. If it proves a success the names of Pearson and Gilliland, its first advocates, will be immortalized in history.

In conclusion, I wish to congratulate the members present on the high position this Association occupies in the list of State associations, and especially on the election of our Secretary at the Minneapolis meeting to the position of Secretary of the American Veterinary Medical Association, which gives this Association additional prestige in the national and international affairs of the profession.

I request of each of you a renewed spirit of loyalty to our Association and all its interests, and wish you all health, happiness, and success throughout all future years.

The Secretary read his report, as follows:

SECRETARY'S REPORT.

MR. PRESIDENT AND MEMBERS: There has been encouraging progress in Association affairs since our last meeting.

Correspondence with the members indicates a commendable interest in the welfare of our organization and in the profession as a whole. The members have been prompt in responding to requests for assistance in getting up a programme, and in otherwise preparing for the meeting. A hopeful feeling has been generated by the prosperous state of veterinary practice, and attention to business has, fortunately, not led to such narrow and selfish views on the part of the members as would cause them to neglect or shoulder upon others the broad interest of the profession at large, the interests of which can be cared for only by such an organization as ours. It is evident to me that our members are almost without exception thoroughly awake to the great benefits which they may derive personally from their membership, and to those which are bestowed upon the profession at large through the influence of our State Association.

The work of the Secretary is considerable in amount and exacting in character, but I have been much gratified by seeing on every hand a reward in the form of good results from labor bestowed.

We now have an active membership of 74. Only 5 of these have allowed themselves to fall into arrears in dues beyond the limit set by the By-laws. The remainder have manifested their interest in the affairs of the Association by ready payment of dues. Collections have as a rule been very satisfactory. Inasmuch as the annual dues are so small, and as it is manifest that the Association requires all the money it can raise through legitimate means for its operation, it is cause for wonder that members should allow themselves to fall into arrears so far as to make them subject to suspension. If a member has not been able to sustain his interest in the Association, the logical and proper thing for him to do would be to submit his resignation and thus free himself from all obligation. Those who are in arrears have been notified three times within the last six months and the consequence of continued delinquency has been detailed to them. There is nothing left for me to do but advise that they be suspended from membership for non-payment of dues.

I would call attention to the fact that it is the policy of the Association to put upon the list of honorary members not subject to dues any members who may remove from the State, or who may go into some other honorable profession or business, provided they are in good standing at the time of cessation of active membership and their dues are paid up. It is very much desired that members thus making a change will keep the Secretary informed in regard to it so that the Association may take appropriate action.

At the last meeting of the Association 36 members were suspended for non-payment of dues. These delinquents had accumulated during a period of ten years, and it was thought the interests of the Association demanded that such action should be taken. The Secretary was directed to send a letter to each one of these, apprising him of the action taken and enclosing a statement of account. This was done at two different times in the cases of 23, whose whereabouts could be ascertained; the remainder either had died or their addresses had been lost. One of the 23 has since died, and of the remaining 22 one has paid up his arrears and asked for reinstatement to active membership. A few others sent replies which will be referred to at another time. Of 39 others who had been suspended for non-payment of dues during the previous history of the Association, 17 were still alive and their addresses were obtainable. A letter and statement of dues was sent to each one of these on two different occasions. One responded by paying up arrears and asking for reinstatement; two others showed their interest by making replies which will be referred to later. It is wellnigh impossible to get any sort of reply from more than a few of those suspended members, much as it is desired. Unless some action which they may view with more favor is taken by the Association I think it would be justifiable to discontinue efforts to bring them again into relations with the Association.

To every veterinarian in active practice in the State who is not a member, but who is eligible to membership, I sent a letter asking him to submit his application. Up to this time 17 have responded, and others are expected to do so during this meeting. This is very encouraging; but still there are about sixty veterinarians in the State who ought to join with us in active membership. It is the duty of every member to see that this result is accomplished, and I hope that a long stride may be taken in this direction during the coming year. I wish all would realize how easy it would be for us to have in Iowa a veterinary association which had developed all the strength which the union of all the veterinarians in the State into such organization would give. Time and space will not allow me to go into detail, as I should like, on this subject, but let us all strive to reach the ideal in regard to membership to which I have just referred.

In the interest of the veterinarians of the State I took up the matter of subscriptions to the veterinary journals. By consulting the subscription lists I found that there were only 68 subscribers in Iowa to one or both of the American veterinary journals. Out of these 68, 22 are non-graduates. In other words, 33 per cent. of the 68 subscribers are non-graduates. This, it must be confessed, is very complimentary to the non-graduates, but not very flattering to the graduates. This left 94 graduates in the State who were not subscribers. A large percentage of these are members of this Association. To each of these an offer of a special subscription price was sent. I am much pleased to say that up to this time 9 have taken advantage of this offer and 3 of these become subscribers to both journals. It is hoped that many more will avail themselves of this offer before it is withdrawn, January 16th. Still, I fear that very, very much will be left to be desired. I should state that one veterinarian wrote me that he is receiving a journal in another's name, and another that he was getting an issue monthly through a newsdealer. I am glad to be able to report that no members have died since our last meeting.

Whether speaking for myself or for my successor, I would request that each member write the Secretary at least one letter each year.

Respectfully submitted,

John J. Repp,
Secretary.

The Treasurer submitted the following report:

TREASURER'S REPORT.

*Receipts.*

| | | |
|---|---|---|
| Cash on hand Feb. 10, 1902, to Jan. 12, 1903, . . | $66.30 | |
| Cash for dues Feb. 11, 1902, inclusive, . . . | 62 00 | |
| Cash for membership fees Feb. 11, 1902, to Jan. 12, 1903, inclusive,. . . . . . . . . | 42.00 | |
| | | $170.30 |

*Disbursements.*

| | | |
|---|---|---|
| Cash refund of membership fee to W. L. Evers, . | $2.00 | |
| Cash to H. E. Talbot account Clinic fourteenth annual meeting, . . . . . . . . . | 10.00 | |
| Cash refund of membership fee to J. V. Jewell, . | 2.00 | |
| Cash refund of membership fee to T. D. Hulme, . | 2.00 | |
| Cash Secretary's allowance, . . . . . . | 25.00 | |
| Cash to Secretary for editing and typewriting Proceedings Fourteenth Annual Meeting, . . . | 20.00 | |
| Cash to J. Miller for postage, . . . . . . | .53 | |
| Cash for 9 registered letters, . . . . . . | .72 | |
| Cash for letter copy book, . . . . . . . | 1.50 | |
| Cash for letter copy book, . . . . . . . | 1.50 | |
| Cash for badges and express on same, . . . | 9.25 | |
| Cash for express, . . . . . . . . . | 1.20 | |
| Cash for stamps, . . . . . . . . . . | 18.75 | |
| Cash for printing and stationery to Ames *Times*, . | 22.25 | |
| Cash for printing and stationery to Hodson Bros., . | 5.50 | |
| | | 122.20 |
| Balance in Treasurer's hands Jan. 12, 1903, . . | | $48.10 |

Respectfully submitted,

JOHN J. REPP,
Treasurer.

The following committee was appointed to audit the Treasurer's accounts: Drs. J. Miller, J. S. Potter, and Hal C. Simpson.

The auditing committee made the following report:

We, the auditing committee for the fifteenth annual meeting of the Iowa State Veterinary Medical Association, hereby certify that we have examined the above account of the Treasurer, and that we find it correct.

HAL C. SIMPSON,
J. S. POTTER,
J. MILLER,
Auditing Committee.

By vote of the Association the report was accepted.

The President appointed A. A. Adamson a member of the Board of Censors in place of W. H. Austin, who was absent.

The Board of Censors reported favorably upon the following applicants for membership: Robert J. W. Briggs, Garner; F. H. Thompson, Des Moines; F. R. Ahlers, Lamotte; C. O. Van Winkle, Salem; A. F. Baldwin, Des Moines; C. J. Heckard, Wheatland; Gustave A. Kay, Avoca; Walter A. Stuhr, Ames; A. L. Wood, Hampton; O. R. Moyer,

Cedar Rapids; F. N. Elwell, Bancroft; S. P. Talbot, Oskaloosa; C. A. Bradley, Marion; J. Brodie, Cedar Rapids; D. Barrett, Cascade; L. L. Diller, Grundy Centre; G. W. Blanche, Belle Plaine; J. E. Frank, Sandyville.

Report of Board of Censors was received by vote.

On motion, the rules were suspended and the Secretary instructed to cast the ballot of the Association for those whose names were read. This was done, and the President declared them duly elected.

The Board of Censors reported favorably upon the following for reinstatement to active membership: J. W. Scott, Manchester; J. E. Harrison, Burlington; J. G. Parslow, Shenandoah.

The report of the Board of Censors was received by vote.

On motion, the rules were suspended and the Secretary instructed to cast the ballot of the Association for those whose names were read. This was done, and the President declared them duly reinstated.

Dr. T. A. Shipley, Inspector Bureau of Animal Industry, invited the members to visit the Sinclair Packing Plant between 1 and 2 P.M.

Mr. Thos. H. Simmons, Secretary of the Commercial Club, invited the members to attend the Poultry Show, admission to be secured by the badges.

Adjournment for lunch at 12 M., to meet at 2 P. M.

*Afternoon Session.*

Meeting called to order at 2.30 P.M. by President Gibson.

The Committee on Disease and Treatment reported as follows:

REPORT OF COMMITTEE ON DISEASE AND TREATMENT.

Your committee beg leave to report briefly. We have nothing new nor startling to offer, but only a review and a few minor suggestions, and hope our effort may be the means of bringing out a good discussion if nothing more. Throughout our State domestic animals have enjoyed particularly good health. There has been no general epizootic. Many localities have been visited by local diseases of different kinds, but they were soon brought under control. Probably we have had more ergotism than for several years past owing to the excessive moisture, rank growth, and lack of sunshine. Almost every pasture was more or less affected; especially was the ergot abundant on the blue grass, red top, and similar grasses.

There was a great deal of enzootic ophthalmia among cattle, especially in the southern part of the State. The loss to stockmen comes principally from shrinkage of animals affected. Treatment is impracticable, especially in large herds.

Rabies has been quite common during the year just closed. We are of the opinion that if farmers would enforce the trespass law throughout the State and prohibit hunting on their farms and in the public highways, the different breeds of hounds and hunting dogs would soon diminish. Then, instead of the general fifty-cent tax, put on a tax so high that only the very best dogs would be worth keeping, and as a result we would soon have the periodical attacks of rabies reduced to a minimum. This means of handling rabies would be beneficial to the sheep interests of the State.

There seems to be very little trouble in feeding cornstalks this year. Why is this? Is it due to the rank growth of the stalks, the excessive moisture, the large amount of grasses along fences and ravines, and abundance of water in every ditch?

There has been considerable sheep-scab in the sheep-raising districts, but sheep-owners do not talk much of this disease when they get it. Almost every farmer who owns a flock worthy the name is prepared to handle this disease, as he has convenient dipping tanks, while any of the Government dips will eradicate the trouble if directions are followed.

We have had the privilege of examining several flocks of sheep which were shipped in that developed scab of the worst form after being inspected and dipped at the dipping stations on entering the State, and know of many more where no veterinarian was called. The best plan is to advise feeders to buy natives, even at a little higher price, as their profits would be more in the end and more satisfactory.

Tuberculosis is a disease that probably none of us will ever see blotted out of our State. Our interstate laws and regulations seem to be insufficient to cope with this or sheep-scab, as the outbreaks seem to be more frequent where either cattle or sheep are imported into the State from the ranges. Perhaps the Bang method is the only practicable method of combatting this disease without entailing too great a hardship on the stock-owners.

Hog cholera is prevalent in a few sections, but not nearly as much so as in former years of bumper corn crops. There are large areas in Southern Iowa and Missouri, where land has been farmed for over sixty years, and hogs have been raised almost continuously, where they never had hog cholera or swine plague. Is it due to mineral deposits, the water, or the blue grass pasture and mast in their pastures? If not, why? Speaking of radical cures for a stock evil, a very successful sheep-breeder remarked to us once that the only way to make a success of the sheep business was to buy the best long-range gun possible, load shells heavily with buckshot, shoot so that there will be no report or howl, only that of the gun, and keep your mouth shut. The report was fully discussed.

The Judiciary Committee made the following report:

### REPORT OF THE JUDICIARY COMMITTEE.

We, the Judiciary Committee of the I. S.V. M. A., after a careful inquiry, find that Dr. S. H. Johnston, of Carroll, Iowa, a member of this Association, has been guilty of non-professional conduct in that he has assisted in instituting and promoting a corresponding school of veterinary science for the express purpose of teaching veterinary science to farmers, breeders, and non-graduate veterinary practitioners. We, therefore, recommend that he be expelled from membership in the Iowa State Veterinary Medical Association.

J. I. Gibson,
J. R. Sanders,
P. Malcolm,
Wm. Drinkwater,
John J. Repp.

The report was adopted.

On motion, Dr. S. H. Johnston was expelled from membership in the Association by a unanimous rising vote.

New business was then taken up.

The resignations of T. A. Bown, Chariton, and M. Y. Schaffer, Des Moines, were accepted.

On motion, it was voted to receive applications for reinstatement to membership from all those suspended for non-payment of dues, provided they pay the sum which they were in arrears at the time when they were made subject to suspension according to Article IV. of the By-laws.

Dr. Bauman reported the following cases: "Rupture of Rectum and Vagina in a Mare during Parturition; Successful Treatment." "Obstruction of Bowels in a Horse Due to Abscess in Lumbosacral Region."

Adjournment was taken at 6.30 P.M. to 7.30 P.M.

*Evening Session.* The meeting was called to order at 8 P.M. by President Gibson.

Dr. S. H. Bauman presented a paper entitled, "Observation on Country Practice."[1]

Dr. J. W. Scott presented a report of a case entitled, "Embolism of the Pulmonary Artery in a Horse."[2]

Dr. Sanders read a report of "Three Anomalies Met with in Castration."[3]

Dr. Drinkwater read a report of "Fracture of the Ribs in a Horse."[4]

Dr. A. S. Brodie read a report upon "Five Cases of Azoturia."[5]

Dr. Malcolm made an extemporaneous report of an "Autopsy on a Calf."

Dr. McLeod read the following reports:

"The Use of Antistreptococcus Serum in Purpura Hemorrhagica."[6]

"Results of Four Operations for Cribbing."[7]

### SECOND DAY—*Morning Session.*

#### CLINIC AND JUDGING.

The clinic was under the supervision of Dr. J. W. Griffith, assisted by Drs. T. A. Shipley and O. R. Moyer. The following operations were performed: Castration of cryptorchid, Dr. G. A. Scott, Independence; stringhalt, Dr. F. F. Parker, Oskaloosa; cribber, Dr. J. H. McLeod, Charles City; oöphorectomy on bitch, Dr. J. H. Potter, Iowa City; trephining for removal of tooth, Dr. J. W. Scott, Manchester; canker of foot, Dr. E. A. Buxton, Vinton; oöphorectomy on bitch, Dr. W. A. Heck, Maquoketa; castration of cryptorchid, Dr. C. E. Stewart, Chariton; oöphorectomy on bitch, Dr. J. H. McLeod, Charles City.

Dr. G. A. Johnson gave a demonstration in connection with some pathological specimens collected at the Sinclair Packing Plant by Dr. T. A. Shipley. The specimens were as follows: Tuberculosis of liver of ox, multiple abscess of liver of ox, cryptorchid testicles from swine, parenchymatous mastitis of cow, cystic ovaries from sows, generalized tuberculosis of hog, cholera of hog, fibroma of larynx of cow.

An exercise in horse judging, arranged by the local committee, took place between 11 and 1 o'clock. Five classes were exhibited. The members first judged the horses, and each selected what he considered the best. Then a committee, consisting of Drs. J. I. Gibson, P. O. Koto, and H. E. Talbot, made the final decisions. The following were the winners in the respective classes:

Draught Team. First, Standard Oil Company; second, J. H. Stein Transfer Company.

[1] See page 144.

[2] See February number, page 106.

[3] See page 164.

[4] See page 168.

[5] See February number, page 109.

[6] See page 166.

[7] See page 167.

Carriage Team for Action. First, W. A. Dobson ; second, H. R. Shafer.

Carriage Team for Conformation. First, W. A. Dobson ; second, H. R. Shafer.

Single Driver for Action. First, W. A. Dobson ; second, H. R. Shafer.

Single Driver for Conformation. First, W. A. Dobson ; second, H. R. Shafer.

The exhibitors are dealers in high-class horses, and the members had the opportunity of studying some of the best horses to be found in America.

Dr. J. W. Griffith is to be congratulated upon his success in getting the clinic started at 8 A.M., and in having both clinic and judging completed by 1 P.M.

*Afternoon Session.* The meeting was called to order at 2 P.M. by President Gibson.

J. W. Scott was appointed a member of the Committee on Resolutions to take the place of C. A. Clinton, who was absent.

Dr. Adamson read a paper entitled, "Compressed Air in the Treatment of Fistulas and Ulcers."[1]

Dr. J. Miller read a paper entitled, "Gastroenterotomy, with Report of a Case."[2]

Dr. G. L. Buffington read a paper entitled, "Ulcerative Enteritis in the Horse."[3]

Dr. W. A. Heck read a report upon "Three Cases Showing the Use of Oil of Turpentine in the Treatment of Atrophy of the Shoulder Muscles."[4]

Dr. T. A. Shipley made a report of the Committee on Sanitation as follows:

REPORT OF COMMITTEE ON SANITATION.

MR. PRESIDENT: Your Committee on Sanitation, or rather its Chairman, after vain appeals to other members of the committee and other members of the profession in the State for help, desires to submit its last year's report to be read by title at this time.

Our reason for so doing is partly because it seems as applicable to conditions to-day as it did one year ago. Not that there has not been any good and honest sanitary work done by veterinarians, but what has been done has evidently been done along the quiet avenues of education rather than the more conspicuous and bustling ones of legislation, and this work can probably be best brought out by a sort of old-time experience meeting, in which each member here may give briefly his knowledge of local conditions and his efforts to right them, and his success or failure and the reasons he assigns for such success or failure. Each is equally valuable as a criterion for future action. And for this reason also we wish to cut the report short so as to give what extra time there may be for this discussion.

But we want to again urge on you the especial need of local meat and milk inspection in cities of 10,000 inhabitants and upward, for, despite the large amount of Federal inspected meat sold in all the markets, the cities of this size naturally become the dumping ground of all undesirable animal carcasses and products that would obviously not pass our Federal inspectors, and because butchers and scalpers in smaller places do not have the oppor-

[1] See page 146.

[2] Will be published in April number.

[3] Will be published in April number.

[4] See page 169.

tunity to get this class of stuff on the markets without the publicity which would kill their nefarious traffic. But in places of 10,000 inhabitants and upward there can always be found some who are willing and able to handle questionable goods without being found out in a way that they can be brought legally to task for their transgressions, or that publicity given their operations which would effectually check them. The only real solution of this problem seems to be for cities of this size to have local city inspection at a public abattoir, and a system of insurance for clinically sound animals, for it is, of course, tuberculosis in this class of animals that would cause the heaviest losses, and they should not be altogether borne by the slaughterer, but, wherever possible, the loss should be made to revert to the original producer. It is this problem that we desire to emphasize and have discussed, because we believe it will lead to the detection of centres of this and other maladies, and will do much to educate the people to view the work of the sanitarian as a help to their progress rather than as a hindrance.

The State Veterinarian, the executive sanitary officer of the State, reports an abundance of routine work with no very serious menace to livestock interests such as has gained a temporary foothold in the New England States. But even such outbreaks tend to emphasize the importance of the sanitarian and will, perhaps, lead to better results than were anticipated.

Another important point which we wish to emphasize at this time is the care the veterinarian should exercise in examining dirty-nosed horses and the importance of educating horse-owners of the dangers from glandered horses. Recent veterinary literature has chronicled the death of two brothers in an adjoining State from this malady after having been treated by their family physician for smallpox, and the infection of a woman physician who made the bacteriological investigation in the foregoing cases. If science would reveal to us a germ—as virulent a germ as this one—with which we could inoculate the general apathy on sanitary matters, the problem would be solved. The latent enthusiasm thus brought into play could not be checked in its good work.

Respectfully submitted,

T. A. SHIPLEY,
Chairman.

The Board of Censors reported favorably upon the following applications for membership: Carl W. Gay, Ames; W. E. Miller, Cherokee; Ralph F. Graham, Colfax, and C. G. Martin, Des Moines.

On motion, the rules were suspended and the Secretary instructed to cas the ballot of the Association for those whose names had been read. This was done and they were declared duly elected.

Dr. Louis A. Klein, of Fort Worth, Texas, was elected to honorary membership.

By vote of the Association, the following members were suspended for non-payment of dues: J. J. Moore, Lamoni; H. Shipley, Sheldon; R. C. Sayers, Fairfield, and J. O. Simcoke, Davenport.

On motion, ten dollars was appropriated to J. W. Griffith to defray expense of clinic.

On motion, twenty dollars was voted to the Secretary for editing, typewriting, and having printed the proceedings of the meeting.

The Committee on Resolutions reported as follows:

Mr. President and Members: Your Committee on Resolutions begs leave to report as follows:

Whereas, It has been the experience of all institutions of learning that in order to accomplish the best results it has been necessary that each division should have at its head a man highly qualified in the knowledge of that particular branch of science; and

Whereas, The Deanship of the Veterinary Division of the Iowa State College remains vacant; be it

*Resolved*, That we, the members of the Iowa State Veterinary Medical Association, in annual session assembled, would respectfully request that the Board of Trustees of that institution place a veterinarian in the position of Dean of the Veterinary Division of that College at an early date.

*Resolved*, That we, the Iowa State Veterinary Medical Association, learn with much pride and approbation the good results already obtained by Drs. Pearson and Gilliland, of Pennsylvania, in their researches upon the subject of vaccination of cattle against tuberculosis; and be it further

*Resolved*, That we offer them our encouragement in the attempts they are now making to render their method of vaccination practical for use in the herds of our country.

*Resolved*, That we, the Iowa State Veterinary Medical Association, express our strong disapproval of correspondence schools of veterinary science, whose purpose it is to give instruction in veterinary science to farmers, breeders, non-graduate veterinary practitioners, and others who are not graduate veterinarians; and be it further

*Resolved*, That we also express our strong disapproval of the instruction of non-graduates in any other than the regular undergraduate course in any veterinary college.

*Resolved*, That we endorse the holding of a clinic at each annual meeting, and that live-stock judging be conducted as a school of instruction in the science of judging at each annual meeting, and that the Committee on Clinics be hereby authorized to furnish suitable score-card blanks for the use of the members in recording their judgment, with reasons therefor.

*Resolved*, That we extend our thanks to the local committee, consisting of Drs. J. W. Griffith, T. A. Shipley, and O. R. Moyer, for the courteous entertainment and excellent clinic furnished by them.

*Resolved*, That we tender our thanks to the citizens, and especially to the city officials, of Cedar Rapids for their kind hospitality.

*Resolved*, That we tender our thanks to the Commercial Club of Cedar Rapids for having placed a convention hall free of charge at our disposal.

G. A. Johnson,
F. J. Neiman,
J. W. Scott,
Committee.

The report was adopted by vote.

Dr. J. H. McLeod offered the following resolution:

*Resolved*, That we, the Iowa State Veterinary Medical Association, offer a special vote of thanks to the management of the Grand Hotel of Cedar Rapids for the uniformly courteous and satisfactory treatment which they have accorded to our members throughout our sessions. Adopted by vote.

A volunteer paper was read by Dr. J. W. Scott entitled, "Principles of Heredity."

The following officers were elected for the ensuing year:

*President:* Dr. T. A. Shipley, Cedar Rapids.

*Vice-Presidents:* Dr. C. E. Stewart, Chariton, and Dr. F. J. Neiman, Marshalltown.

*Secretary and Treasurer:* John J. Repp, Ames.

*Board of Censors:* Drs. P. Malcolm, New Hampton; J. H. McLeod, Charles City, and H. C. Simpson, Denison.

Absolute harmony prevailed in the election. There were no opposing candidates for any office, and in each case the rules were suspended on motion, and either the President or the Secretary instructed to cast the ballot of the Association.

On vote of the Association, the resignation of Dr. G. A. Johnson from membership was accepted.

The following special resolution was adopted.

*Resolved,* That it is the sense of the Iowa State Veterinary Medical Association that each member personally investigate the method by which each non-graduate veterinary practitioner obtained his certificate of registration, including the question as to whether his vouchers were reputable stock-owners and freeholders; also that each member prosecute any who are now practising veterinary medicine, surgery, or dentistry in Iowa without proper registration with the Board of Veterinary Examiners.

Dr. Simpson moved that the Committee on Diseases and Treatment be instructed to investigate azoturia during the coming year and report upon it at the sixteenth annual meeting.

Motion adopted by vote.

On motion, the Association adjourned to convene in sixteenth annual meeting at Des Moines at the call of the President and Secretary.

The following members were in attendance: Drs. A. A. Adamson, Newton; S. H. Bauman, Birmingham; G. W. Blanche; Belle Plaine; C. A. Bradley, Marion; A. S. Brodie, Cedar Falls; J. L. Brodie, Cedar Rapids; G. L. Buffington, Baxter; E. A. Buxton, Vinton; William Drinkwater, Monticello; F. H. P. Edwards, Iowa City; J. I. Gibson, Denison; Ralph F. Graham, Colfax; J. W. Griffith, Cedar Rapids; J. E. Harrison; Burlington; S. K. Hazlet, Oelwein; W. A. Heck, Maquoketa; C. J. Heckard, Wheatland; G. A. Johnson, Sioux City; G. A. Kay, Avoca; G. S. Kerr, Washington; S. H. Kingery, Creston; P. O. Koto, Forest City; J. H. McLeod, Charles City; J. H. McNeall, Ames; P. Malcolm, New Hampton; C. G. Martin, Des Moines; J. Miller, Ottumwa; W. E. Miller, Cherokee; O. R. Moyer, Cedar Rapids; F. J. Neiman, Marshalltown; F. F. Parker, Oskaloosa; J. S. Potter, Iowa City; John J. Repp, Ames; James E. Robertson, Monona; J. R. Sanders, Corydon; George A. Scott, Independence; J. W. Scott, Manchester; T. A. Shipley, Cedar Rapids; Hal C. Simpson, Denison; C. E. Stewart, Chariton; H. E. Stewart, Lacona; H. E. Talbot, Des Moines; George M. Walrod, Storm Lake.

The following visitors were in attendance:

Peter Boyd, Cedar Rapids; William Bryant, Marion; C. Carney, City Board of Health, Cedar Rapids; Chief Cook, Fire Department, Cedar Rapids; Zan Cotter, Chicago, Ill.; D. Cushman, alderman, Cedar Rapids; W. A. Dobson, Marion; J. C. Douns, Vinton; Dr. W. L. Evers, Iowa Falls; W. Garretson, White Lake, S. D.; C. Ham, Solon; W. N. Hake, Vinton; James Hughes, alderman, Cedar Rapids; Charles D. Huston, Mayor,

Cedar Rapids; C. B. Hamilton, Cedar Rapids; Dr. Lawler, city physician, Cedar Rapids; John Limbach, City Board of Health, Cedar Rapids; Dr. R. Mollance, Reinbeck; Mrs. John J. Repp and two sons, Ames; J. Robertson, Norway; C. R. Riley, Cedar Rapids; Thomas H. Simons, Secretary Commercial Club, Cedar Rapids; L. J. Strong, Iowa City.

Respectfully submitted,

JOHN J. REPP,
Secretary.

---

### CONNECTICUT VETERINARY MEDICAL ASSOCIATION.

THE annual meeting of the Association was held at Hotel Hartford, Hartford, Tuesday, February 3, 1903. The meeting was called to order at 2.30 P.M. by First Vice-President, Dr. H. Whitney. The following members were present: Drs. E. C. Ross, J. H. Gardner, H. Whitney, H. E. Bates, J. E. Underhill, L. B. Judson, George T. Crowley, F. G. Atwood, P. F. Finnegan, F. A. Ingram, and B. K. Dow. Visiting veterinarians: Drs. Charles L. Cotton and C. E. Dornheim.

The reports of the Secretary and the Treasurer were read and voted to be accepted as read.

There not being a quorum of the Board of Censors present, upon motion of Dr. Ross, it was voted that the President appoint members present to fill the vacancies.

The President appointed Drs. L. B. Judson and J. E. Underhill.

The Secretary presented the following veterinarians' applications for membership in the Association: George W. Loveland, of Torrington, C. V. C., '94, member A. V. M. A.; Fred F. Bushnell, of Winsted, New York State V. C., '02; C. E. Dornheim, of New London, C. V. C., '02, and Charles L. Cotton, Vet. Dept. U. of Pa., '01.

These names were referred to the Board of Censors for their action.

The Board reported they found all applicants eligible, and recommended that they be elected to membership in the Association.

It was voted to suspend the By-laws, and the applicants were unanimously elected to membership.

The following officers were unanimously elected to serve for the ensuing year:

*President.*—Dr. Harrison Whitney, of New Haven.

*Vice-Presidents.*—Dr. Thomas Bland, of Waterbury, and Dr. J. E. Underhill, of New London.

*Secretary.*—Dr. B. K. Dow, of Willimantic.

*Treasurer.*—Dr. E. C. Ross, of New Haven.

*Board of Censors.*—Drs. H. E. Bates, of So. Norwalk; F. A. Ingram, of Hartford; R. D. Martin, of Bridgeport; F. S. McGuire, of New Britain, and L. B. Judson, of Winsted.

The Secretary brought up the subject of having the meetings begun earlier in the day, and material provided for a good clinic in connection with it.

The matter was discussed at some length by most of those present.

Dr. Ross extended a cordial invitation to the members to hold the next meeting in New Haven at his hospital, and he would have material ready for clinics and operations.

Dr. Atwood also extended an invitation to the members to visit his hospital and use his new operating table.

It was voted to hold the semi-annual meeting at Dr. Ross' hospital, in New Haven, August 18, 1903, at 10 A.M.

Upon motion, made by Dr. Ingram, seconded by Dr. Colton, it was voted that the following New Haven veterinary surgeons act as a committee to provide ample material for a clinic, and to select operators and make all necessary arrangements for the meeting: Drs. E. C. Ross, H. Whitney, F. G. Atwood, and J. H. Kelley.

Upon motion made by Dr. Atwood, seconded by Dr. Ingram, it was voted that the Committee of Arrangements extend a special invitation to Dr. Thomas Bland, of Waterbury, to be present at the next meeting and perform one or more operations.

The Secretary presented the names of Drs. R. P. Lyman and N. S. Mayo; both having moved from the State, wished to resign their membership in the Association.

Dr. Ross said these two members had done a great amount of work for the Association, and were very instrumental in its reorganization, and thought, as a matter of courtesy and respect to these two members, their names ought to be placed on the roll of honorary membership in the Association, and made a motion to that effect. After considerable discussion, the motion was lost.

Dr. F. G. Atwood read a paper on "Technique of Veterinarians' Operating Rooms and Equipment." He also exhibited several photographs of his new operating-table, and explained its workings and advantages.

Dr. B. K. Dow read a paper on "Cerebro-spinal Meningitis," which elicited much discussion and gradually drifted into a lively and interesting discussion of glanders and tuberculosis.

The hour being so late, it was decided to leave the subjects for discussion go over to the next meeting. The subjects were: "Azoturia," "Canine Distemper," and "Scrotal Hernia."

The meeting adjourned at 7 o'clock.

B. K. Dow,
Secretary.

---

### CENTRAL CANADA VETERINARY ASSOCIATION.

At a well-attended meeting of the veterinary practitioners of the city of Ottawa and vicinity for the purpose of perfecting arrangements for the entertainment of the American Veterinary Medical Association at its fortieth annual meeting, to be held during the first week in September next, the Central Canada Veterinary Association was launched.

Through the courtesy of the city, which is doing everything in its power to make the coming meeting of the A. V. M. A. a grand success, this meeting of the veterinarians of Ottawa and vicinity was held in the City Hall on Thursday evening, February 5th.

Those present were: Drs. Higginson, Rockland; Lynchke, Carp; McGuire, Cornwall; Young, Cobden; Young, Merrickville; Young, Almonte; Allen, Brockville; Irvine, Maxwell; Rutherford, Harris, White, James, Higgins, Hollingsworth, Hopkins, and Boucher, of Ottawa.

The meeting was called to order by Dr. A. W. Harris, chairman of the previous meetings held by the city practitioners. Dr. W. W. Boucher read the minutes of the previous meetings that those attending might become acquainted with the steps already taken toward a concerted action for the entertainment of the A. V. M. A.

Dr. J. G. Rutherford explained in detail his visit to Minneapolis, at which time he invited the A. V. M. A. to hold its next annual meeting at the Canadian capital, the proposition being heartily supported and unanimously received by them. He also explained the present status of the veterinary profession in Canada, and, that with the exception of Manitoba, where there has been an exceedingly strong Association for fourteen years, which Association has been the means of getting through the Legislature of that Province the strongest bill regulating the practice of veterinarians enjoyed by any such body in the world; and Quebec, where an Association was formed last year which succeeded in obtaining legislation, and who are this year endeavoring to strengthen their existing legislation.

Dr. Rutherford explained that, while an Association here in this section would be unable to take active steps in obtaining legislation, its salutary influence would be beneficial in stimulating and encouraging the existing veterinary associations in both the Provinces of Ontario and Quebec to a more concerted action.

Active discussion followed, in which each veterinarian present took an active part, all agreeing that the time was opportune for effective work toward organization and the stimulation of a more friendly feeling among the members of the profession.

Dr. Rutherford moved that we proceed to form an association of the veterinarians of Ottawa and vicinity, to be called "The Central Canada Veterinary Association." The motion was seconded by Dr. White and unanimously carried without further discussion.

After the naming of the Association the next step was the election ot officers, as follows: Honorary President, Dr. J. G. Rutherford, Ottawa; President, Dr. A. W. Harris, Ottawa; Vice-President, Dr. T. A. Allen, Brockville; and Secretary-Treasurer, Dr. W. W. Boucher, Ottawa. After some discussion it was decided that a board of directors was essential, the board to consist of the President, Vice-President, Secretary-Treasurer, and eight additional members to be elected by the Association, the following being unanimously chosen: Drs. Lynchke, Carp; W. C. Young, Almonte; F. Fisher, Carleton Place; W. C. McGuire, Cornwall; A. E. James, Ottawa; G. W. Higginson, Rockland; J. B. Hollingsworth and C. H. Higgins, Ottawa.

It was decided that the Executive Committee should consist of the Ottawa members of the Board of Directors for the sake of convenience in the holding of meetings. Dr. C. H. Higgins was elected as official reporter.

The Executive Committee was directed to prepare the Constitution and By-laws of the Association, to be submitted to the Board of Directors for approval, said Constitution and By-laws to be presented to the next meeting of the Association.

During the discussion relative to the drawing up of the By-laws inquiry was made as to the eligibility for membership in the Association. This matter of eligibility of membership was left wholly with the Executive Committee, and all applications for membership should be passed upon by this body before being presented to the Association.

A motion of thanks was extended to Dr. Rutherford for his service to the profession of Canada in inviting the A. V. M. A. to hold its next meeting in Ottawa and the interest he has taken, not only in the formation of the Central Canada Veterinary Association, but in matters pertaining to the advancement of the profession throughout the whole Dominion. Unanimously carried.

The date of the next meeting was set for Easter Monday evening, April 13, 1903, at 7.30 o'clock, in the City Hall, Ottawa, which, it is expected, will be largely attended by local veterinarians.

On motion of Dr. James, the meeting adjourned.

DR. C. H. HIGGINS,
Official Reporter.

---

### THE VETERINARY ASSOCIATION OF MANITOBA.

THÉ annual meeting of this Association was held in the city of Winnipeg on February 19th, the President, Dr. S. A. Coxe, in the chair. The following members were present: Drs. S. A. Coxe, Brandon; C. Little, Winnipeg; C. McGilvray, Binscarth; J. A. Stevenson, Carman; A. M. Livingstone, Melita; W. A. Hilliard, Minnedosa; W. R. Taylor, Portage la Prairie; H. F. Whaley, Glenboro; W. E. Martin, Winnipeg; W. Harrison, Glenboro; J. Welsh, Roland; W. S. Henderson, Carberry; F. Torrance, Winnipeg; W. J. Hinman, Winnipeg; R. Frame, Treherne; M. B. Rombough, Morden; W. Swinerton, Carberry; W. A. Dunbar; Winnipeg; A. E. Williamson, Winnipeg; E. P. Westell, Winnipeg.

The report of the Secretary-Treasurer and Registrar showed the Association to be in a flourishing condition, the total membership being 77.

The election of officers resulted as follows: President, W. R. Taylor, Portage la Prairie; Vice-President, G. Hilton, Portage la Prairie; Secretary-Treasurer and Registrar, F. Torrance, Winnipeg; Council, Drs. Torrance, Stevenson, Coxe, Hilton, Henderson, and Martin.

A grant of $100 was made to the central committee at Ottawa as a contribution toward the expense of entertaining the A. V. M. A.

Dr. C. D. McGilvray read an interesting paper on "The Physical Examination of the Sick Horse." The essay showed evidence of careful preparation, and the essayist received the thanks of the Association and was awarded the prize offered at the last meeting.

It was decided to hold the semi-annual meeting at Portage la Prairie, the date to be fixed by the Executive.

F. TORRANCE,
Secretary-Treasurer.

---

### A. V. M. A.

OTTAWA, CANADA, SEPTEMBER 1 to 4, 1903.

Secretary Repp, of the Association, has just issued these two very important letters to the members and the Resident State Secretaries:

FEBRUARY 25, 1903.

TO THE RESIDENT STATE SECRETARIES:

I am sending herewith a number of copies of the Constitution and By-laws of the American Veterinary Medical Association, some application blanks and envelopes, and a supply of a circular letter.

You will please, at your earliest convenience, send a copy of the letter to each veterinarian in your State or Territory who is not a member but eligible to membership, and enclose with it an application blank. Reserve the copies of the Constitution and By-laws to send to those who may make a request for a copy. When a copy is sent, Chapters VI., VIII., and XI. should be marked and attention directed to them. In connection with your efforts to secure new members, I would call your attention to Chapters VI. and XI. of the By-laws. Care should be taken not to endorse the application of one who is not qualified by graduation from a recognized school, who is given to unprofessional conduct or bad habits, or who resorts to improper methods of advertising, or uses illustrated letter-heads and envelopes. You are respectfully requested to inquire into these matters before you accept or endorse an application. The initiation fee must be paid in advance, and is best sent direct to me by the applicant when he sends his application, after the application is returned to him by you, or other voucher after endorsement. In some cases a prospective applicant will promise to discontinue practises which render him ineligible if proper explanation is made to him. It is advised that you make such explanation when circumstances seem to warrant it. In all such cases you should write me a statement of the facts, to be filed with the application. The Association is anxious to increase its membership by the addition of all who are eligible, so will appreciate any special effort you may make in this direction.

If the envelope in which the circular and blank are sent is left unsealed, it can be mailed for one cent. In this way a considerable saving in postage can be made.

The very commendable custom of submitting a report at the annual meeting upon matters relating to the veterinary profession in each State and Territory has been firmly established by the State Secretaries. It is hoped that at the coming meeting each State Secretary will be ready with a report. If you cannot be present at Ottawa, and will send your report to me before the meeting, it will be published in the proceedings. There is no more interesting or instructive part of the proceedings than the reports of State Secretaries. You should not overlook this important part of your duties.

The subject of arranging a programme for the Ottawa meeting must at once engage our most serious attention. The contributions should represent every section of the home of our organization and every specialty of our profession. We must have enough papers, and they must be in sufficient variety to engage the attention of all our members. Will you kindly solicit contributions from each one of our members in your State or Territory whom you believe might be willing to take the pains to get up a production of merit, urging him to announce the subject very soon, so that a good programme may be assured at an early date.

It is also very essential that you exert your influence to secure a large attendance for the Ottawa meeting. There are many things to attract our members and others to Ottawa. It will be a delightful trip for anyone, and more so for many whose old homes, which they may wish to revisit, are in the eastern provinces of Canada or in the eastern part of the United States. Rates will be announced in due time. It is expected that the Ottawa meeting will far outclass in every way any that has yet been held, and you can do a very great deal to make it so.

If you will, sometime before or soon after the meeting, send me an itemized bill of your expenses as State Secretary, such bill will be duly audited and paid.

Please inform me of any change of address of any of our members in your State or Territory of which you may become cognizant.

Will you kindly acknowledge the receipt of this letter and package.

I shall be glad to receive from you any advice or suggestions looking toward the advancement of the Association.

Yours respectfully, JOHN J. REPP,
Secretary.

FEBRUARY 23, 1903.

TO THE MEMBERS OF THE AMERICAN VETERINARY MEDICAL ASSOCIATION:

One of the most important things to be done in the way of preparation for the Ottawa meeting is the arranging of a programme. A good programme means a good meeting, and a poor programme means a poor meeting. Whether it be good or bad depends upon you. Your Secretary can only solicit, urge, entreat. He usually has to do all of these and more. Making a programme is always difficult, and a source of much anxiety to the officers upon whom the responsibility of failure is placed. It should not be difficult. Why not make it easy? The inducements in the way of added prominence given the contributors, the publication of the paper in the proceedings, and the knowledge that help is given to others and something is added to the fund of knowledge should make everyone anxious to contribute.

The programme will be completed at the earliest possible date; by June 1st, if possible. This is the only general solicitation that will be made. If it does not bring the desired result within a reasonable time, the programme will be completed by personal requests to individual members. Please let me have the title of your paper at the earliest possible date. If you are disposed to present a paper, and will let me have your promise to do so, I shall wait a reasonable time for the announcement of your title to follow if you are not decided as yet what it shall be. It is desirable to have contributions which represent every part of the home of our organization and every specialty of our profession.

Please let me hear from you very soon. If you have any suggestion or advice to offer in regard to making the programme, it will be gratefully received.

There is enclosed with this a copy of the revised Constitution and By-laws. Your attention is directed to the proposed amendments, to be found at the end of the booklet, which will be acted upon at the Ottawa meeting.

Yours respectfully,
JOHN J. REPP,
Secretary.

---

President Winchester was a strong advocate of the surgical clinics for the Minnesota meeting. He yearns for the colleges to have a four years' course and the day when the A. V. M. A. will not have to conduct a post-graduate course.

### RESOLUTIONS ADOPTED BY THE KEYSTONE VETERINARY MEDICAL ASSOCIATION.

WHEREAS, Governor Stone has throughout his entire term of office given a careful hearing to our committees, and has carefully considered all matters pertaining to the work of the Veterinary Examining Board and the State Live-stock Sanitary Board; therefore be it

*Resolved*, That this Association hereby place on record its high appreciation of Governor Stone's attitude toward questions pertaining to our profession, and we thank him for his friendly interest and help.

WHEREAS, Our State has received at the hands of our State Live-stock Sanitary Board the greatest benefits possible; and whereas, this work has largely been directed by our colleague and fellow-member, Dr. Leonard Pearson; therefore, be it

*Resolved*, That this Association approve of his reappointment, and that our Board of Trustees be directed to officially take such action as may be necessary to secure his reappointment under the incoming administration.

*Resolved*, That this Association approve the enactment of a law to require that certain infectious diseases be reported to the State Live-stock Sanitary Board.

*Resolved*, That we, the Keystone Veterinary Medical Association, approve of the proposed legislation seeking to secure a re-registration of all veterinarians in the State of Pennsylvania, so that a more accurate record of all practitioners may be made accessible to everyone's interest in the advancement of veterinary education.

*Resolved*, That the Keystone Veterinary Medical Association, now in meeting here assembled, are in hearty accord and hereby pledge our individual and collective support of the Good Roads bill as framed by Senator Sproul, and all kindred legislation as needed to enhance the value of this measure and movement.

